# The role of right ventricular systolic pressure and ARISCAT score in perioperative pulmonary risk assessment

**DOI:** 10.1016/j.bjane.2025.844597

**Published:** 2025-02-17

**Authors:** Yoshio Tatsuoka, Zili He, Hung-Mo Lin, Andrew P. Notarianni, Zyad J. Carr

**Affiliations:** a Yale University School of Medicine, Department of Anesthesiology, New Haven, USA; b Yale Center for Analytical Sciences, New Haven, USA

**Keywords:** Echocardiography, Pulmonary hypertension, Postoperative complications, Risk assessment

## Abstract

**Background:**

Postoperative Pulmonary Complications (PPC) are a significant source of increased morbidity and mortality after surgical procedures. Measures to enhance 30-day PPC risk stratification are an area of significant clinical interest, and integrating common preoperative investigations, such as echocardiography, may enhance quantitative risk prediction when combined with clinical score-based systems, particularly for high-risk populations. The authors hypothesized that Right Ventricular Systolic Pressure (RVSP) would significantly enhance the predictive capabilities of the Assess Respiratory Risk in Surgical Patients in Catalonia (ARISCAT) score in the prediction of 30-day PPC in a Pulmonary Hypertension (PH) study cohort.

**Methods:**

277 patients with the diagnosis of PH, ARISCAT score, and echocardiography-derived RVSP within 12-months of surgical procedure were analyzed. The primary endpoint was the 59-variable 30-day Agency for Healthcare Research and Quality PPC composite. Secondary endpoints included sub composites of Pneumonia (PNA), Respiratory Failure (RF), Pulmonary Aspiration (ASP) and thromboembolic Phenomenon (PE). Adjusted multivariable logistic regression models followed by Receiver Operating Characteristic Curves (ROC) and Area Under the Curve (AUC) analysis were employed to assess the prediction of 30-day PPC.

**Results:**

Mean RVSP was 52.1 mmHg (±17.4). Overall PPC incidence was 29.9%, with RF (19.5%), PNA (12.3%), ASP (5.4%), and PE (3.6%) composites. Logistic regression showed no significant association between RVSP and PPC (Odds Ratio [OR = 1.01], p = 0.307). The ARISCAT score was associated with 30-day PPC risk (OR = 1.02, p = 0.037). Receiver Operating Characteristic (ROC) curve analysis revealed an Area Under the Curve (AUC) of 0.555 for RVSP alone, 0.575 for the ARISCAT score, and 0.591 for the combination of RVSP+ARISCAT for the primary endpoint.

**Conclusion:**

RVSP demonstrated limited efficacy as a standalone predictor of 30-day PPC in patients with PH. Although integrating RVSP with ARISCAT scoring yielded marginal improvements in predictive accuracy, neither metric, independently or in combination, achieved adequate clinical significance for reliable risk stratification. These findings highlight a critical gap in the current preoperative risk assessment for PH-specific predictive tools. Future research should focus on alternative measures that better capture vulnerability to the hemodynamic complexities underscoring PPC in this high-risk population.

## Introduction

Postoperative Pulmonary Complications (PPC) are a significant source of increased morbidity and mortality after surgical procedures. Measures to enhance 30-day PPC risk stratification are an area of significant clinical interest, and integrating common preoperative investigations, such as echocardiography, may enhance quantitative risk prediction when combined with clinical score-based systems, particularly for high-risk populations. Right Ventricular Systolic Pressure (RVSP) is commonly estimated during preoperative Transthoracic Echocardiography (TTE) using the modified Bernoulli equation [RVSP (mmHg) = 4TRV^2^ + RAP, where TRV, Tricuspid Regurgitation Velocity (m/sec) and RAP, Right Atrial Pressure (mmHg)].^1^ Right ventricular systolic pressure is a correlate for right ventricular afterload, and the American College of Cardiology recommends further evaluation of patients with dyspnea and RVSP > 40 mmHg to assess for the presence of Pulmonary Hypertension (PH).^2^ In perioperative settings, PH is associated with significant cardiopulmonary morbidity and mortality.^3^^,^^4^ Given the frequency, economic impact, and increased morbidity of PPC, improving PPC risk prediction is vital for developing preventive perioperative strategies.

RVSP alone and in combination with the Assess Respiratory Risk in Surgical Patients in Catalonia (ARISCAT) score, a validated PPC risk scoring system for general populations, were evaluated to determine if quantitative echocardiographic measures could enhance PPC risk prediction. ARISCAT incorporates factors such as age, preoperative arterial oxyhemoglobin saturation, recent respiratory infection, anemia, surgical site, procedure duration, and urgency to predict in-hospital PPC risk.^5^ While ARISCAT is used to predict in-hospital PPC, recent reports have suggested that its predictive capabilities extend to 30-day PPC.^6^ Acknowledging the risk of 30-day PPC is critically important, as recent reports indicate that the trend toward shorter postoperative length of stays has led to an increase in post-discharge infections and other complications.^7^ The primary objective of this study was to assess whether increasing RVSP alone, or in tandem with ARISCAT predicts 30-day PPC risk in a PH population. The authors hypothesized that Right Ventricular Systolic Pressure (RVSP) would significantly enhance the predictive capabilities of the Assess Respiratory Risk in Surgical Patients in Catalonia (ARISCAT) score in the prediction of 30-day PPC in a Pulmonary Hypertension (PH) study cohort.

## Methods

### Study population

**Figure 1:**
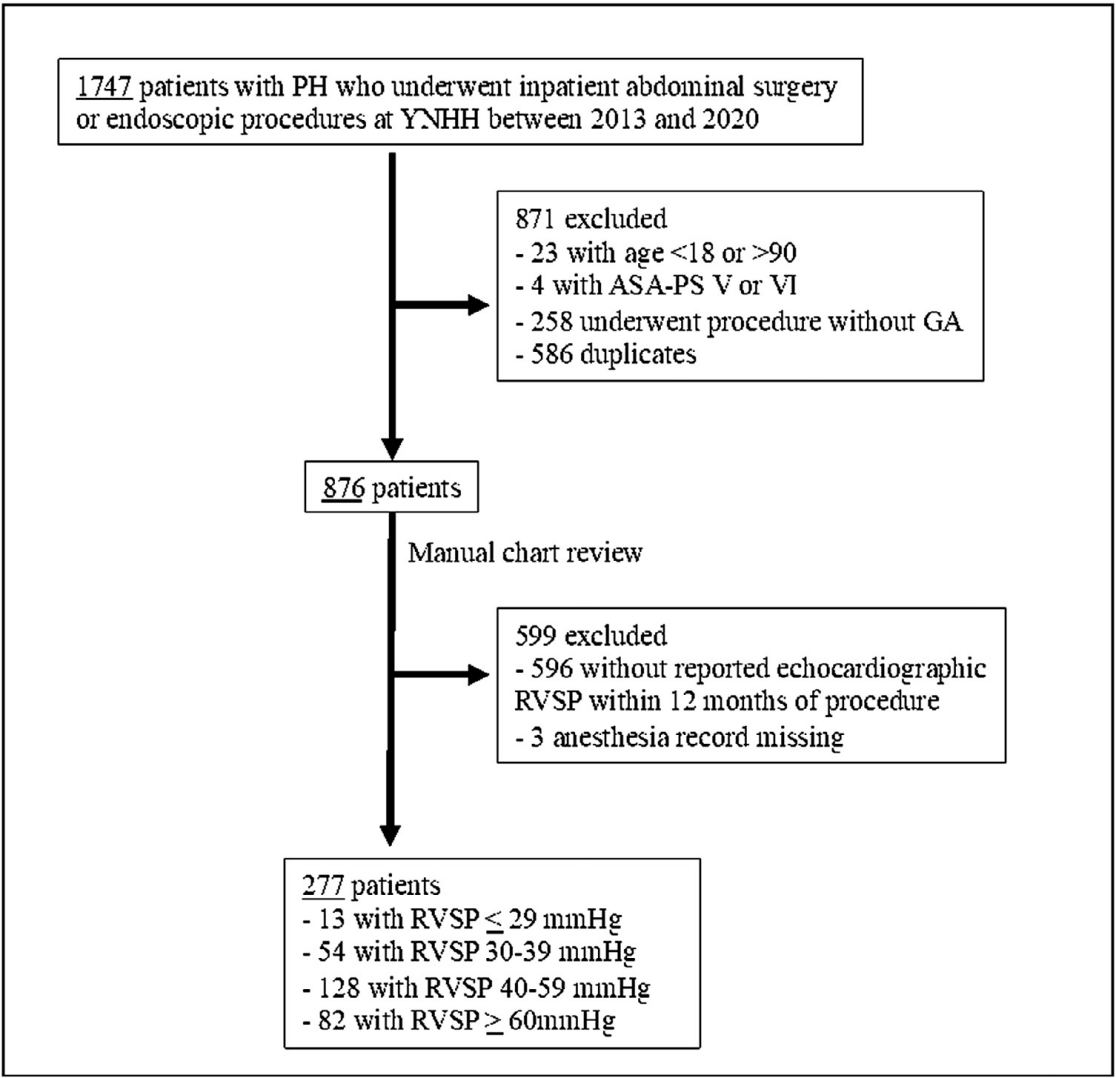
CONSORT flow diagram of patient study inclusion.

This retrospective cohort study was approved by the Yale University Institutional Review Board (IRB#2000032516) with a waiver of informed consent. 1,747 patients with International Classification of Disease version 10 (ICD-10) codes for PH (I27.0, I27.2) who underwent elective inpatient abdominal surgery or gastrointestinal endoscopic procedures at Yale New Haven Hospital between 2013 and 2020 were analyzed. Patients aged < 18 or > 90 years, classified as American Society of Anesthesiologists Physical Status Classification System (ASA-PS) V or VI, and procedures without general anesthesia were excluded. To avoid duplicates in patients with multiple surgical encounters, only the most recent procedure was analyzed. Among 876 eligible patients, 277 with preoperative ARISCAT scores and echocardiographic RVSP within 12-months of surgery were analyzed (Fig. 1).

### Outcomes

Baseline characteristics included age, Body Mass Index (BMI), Elixhauser comorbidities,^8^ and procedure severity, which was classified using the Surgical Outcome Risk Tool (SORT) v2.^9^ The outcomes included 30-day PPC and 30-day mortality. ARISCAT scores were individually tabulated, and PPC was defined using the Agency for Healthcare Research and Quality (AHRQ) PPC composite criteria (Supplementary Table 1). PPC subcategories included Respiratory Failure (RF), Pneumonia (PNA), Aspiration (ASP), and Thromboembolic Phenomena (PE).

### Statistical analysis

Logistic regression analysis was performed to evaluate RVSP and ARISCAT as predictors of PPC and subcategories. Receiver Operating Characteristic (ROC) curve analysis was used to calculate the Area Under the Curve (AUC) for RVSP, ARISCAT, and RVSP+ARISCAT, with and without covariate adjustments using propensity score weighting for age, BMI, Elixhauser comorbidities, and procedure severity. PH patients were further classified into RVSP ≥ 40 mmHg and RVSP < 40 mmHg groups and reanalyzed using similar methods. Only subjects with complete information were included in the analysis.

## Results

### Baseline characteristics

The final cohort included 277 patients (59.9% female) with a mean age of 66.4 years (±14.5), a mean BMI of 29.4 (±8.9), and a median Elixhauser comorbidity count of 7 (Interquartile Range [IQR = 5–8]). Procedural severity was classified as intermediate (62.5%), major (19.9%), and Xmajor/complex (17.7%). The median surgery duration was 92.5 minutes (IQR = 51‒170]. Mean RVSP was 52.1 mmHg (±17.4). Overall, the 30-day PPC incidence was 29.9% (n = 83), and 30-day mortality rate was 3.9% (n = 11). Patients with and without PPC did not differ significantly different with respect to any of the aforementioned baseline characteristics (p > 0.05) (Table 1).

**Table 1:** Baseline characteristics of the studied cohort categorized by presence of right ventricular systolic pressure ≥ 40 mmHg or < 40 mmHg.

Variable	RVSP < 40 (n = 67)	RVSP ≥ 40 (n = 210)	p-value^a^	Overall (n = 277)
Age, in years, median (SD)	62.0 (15.2)	67.8 (14)	0.006^a^	66.4 (14.5)
Gender, Female, number (%)	43 (64.2)	123 (58.6)	0.501	166 (59.9)
Body Mass Index, kg.m^−2^, mean (SD)	29.6 (9.38)	29.3 (8.8)	0.791	29.4 (8.9)
Elixhauser Comorbidities, mean (SD)	6.2 (2.26)	6.4 (1.9)	0.409	6.39 (2)
Procedural Severity, number (%)				
Intermediate	30 (44.8)	143 (68.1)		173 (62.5)
Major	21 (31.3)	34 (16.2)		55 (19.9)
Xmajor/Complex	16 (23.9)	33 (15.7)	0.002^a^	49 (17.7)
RVSP, in mmHg, mean (SD)	32.6 (4)	58.3 (15.3)	<0.001^a^	52.1 (17.4)
ARISCAT score, mean (SD)	26.8 (17.1)	28.5 (16.9)	0.467	28.1 (17)
Surgery duration, in minutes, median (IQR)	140 (89‒229)	82 (49‒144)	<0.001^a^	92.5 (51‒170)
PPC, number (%)	16 (23.9)	67 (31.9)	0.273	83 (29.9)
Infectious pneumonia	7 (10.4)	27 (12.9)	0.729	34 (12.3)
Respiratory failure	10 (14.9)	44 (21.0)	0.339	54 (19.5)
Aspiration	4 (6.0)	11 (5.2)	1	15 (5.4)
Pulmonary embolism	2 (3.0)	8 (3.8)	1	10 (3.6)
Length of stay, in days, median (IQR)	4.9 (3.1‒10.5)	7.71 (4.1‒15.6)	0.013	6.97 (3.7‒13.8)
Thirty-day Mortality, number (%)	3 (4.5)	8 (3.8)	0.81	11 (3.9)

a Student's *t*-test, Chi-Square or Wilcoxon as appropriate. RHC, Right Heart Catheterization; SD, Standard Deviation; RVSP, Right Ventricular Systolic Pressure by echocardiography; ARISCAT, Assess Respiratory Risk in Surgical Patients in Catalonia risk index; PPC, Postoperative Pulmonary Complications; IQR, Interquartile Range.

### Primary endpoint

Adjusted logistic regression analysis showed no association between RVSP and PPC (Odds Ratio (OR) = 1.01, 95% Confidence Interval (95% CI) 0.99–1.02, p = 0.307).

### Secondary endpoints

After adjustment, the ARISCAT score was associated with 30-day PPC (OR_adj_ = 1.02, 95% CI 1.00–1.03, p = 0.037). The ARISCAT score was associated with RF (OR_adj_ = 1.02, 95% CI 1.00–1.04, p = 0.020) but not with PNA (OR_adj_ = 1.01, 95% CI 0.99–1.03, p = 0.348), ASP (OR = 1.0, 95% CI 0.97–1.03, p = 0.981), or PE (OR = 1.01, 95% CI 0.97–1.05, p = 0.594). RVSP was not associated with any of the sub composites, including RF (OR = 1.01, 95% CI 0.99–1.03, p = 0.179), PNA (OR = 1.00, 95% CI0.97–1.02, p = 0.710), ASP (OR = 0.99, 95% CI 0.96–1.02, p = 0.523), or PE (OR = 1.02, 95% CI 0.99–1.05, p = 0.212).

**Figure 2:**
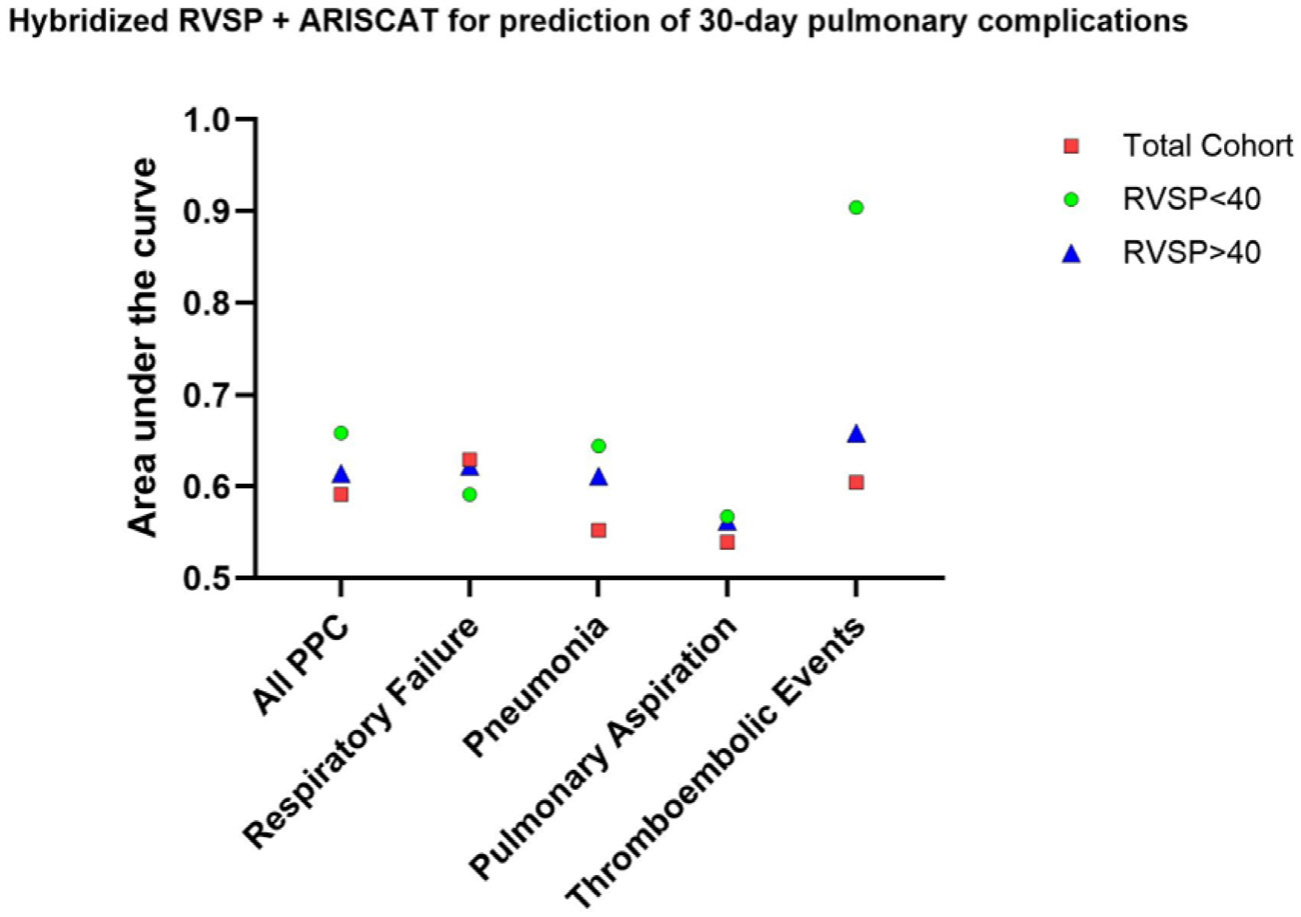
Plot of combined RVSP with ARISCAT score and its predictive performance for composite 30-day postoperative pulmonary complications and relevant sub composites. Although the combination was highly predictive for 30-day thromboembolic events, it requires further validation in a larger cohort. PPC, Postoperative Pulmonary Complications; RVSP, Right Ventricular Systolic Pressure by echocardiography; ARISCAT, the Assess Respiratory Risk in Surgical Patients in Catalonia risk index.

The AUC values were 0.555 for RVSP alone, 0.575 for ARISCAT, and 0.658 for the hybridized RVSP and ARISCAT (Supplementary Table 2). The results remained consistent before and after covariate adjustment. In the subgroup analysis, patients with RVSP ≥ 40 mmHg (n = 210) and RVSP < 40 mmHg (n = 67) did not show a significant difference in PPC (32% vs. 24%, p = 0.273), RF (21% vs. 15%, p = 0.339), PNA (13% vs. 10%, p = 0.729), ASP (5.3% vs. 6.0%, p = 1), PE (3.9% vs. 3.0%, p = 1), or 30-day mortality (4.1% vs. 3.5%, p = 1.0). Similarly, few differences were observed when categorized by RHC (Supplementary Table 3). Hybridized ARISCAT+RVSP consistently outperformed for patients with RVSP < 40, with the exception of RF (Fig. 2).

## Discussion

In summary, RVSP alone was not associated with PPC risk. However, the ARISCAT score, originally validated for general surgical populations and in-hospital pulmonary complications, showed a significant association with 30-day PPC in PH patients. While RVSP enhanced ARISCAT's predictive power for specific subcategories (RF, PNA, and PE), the overall clinical relevance of this improvement was minimal.

Although elevated RVSP remains an important prognostic factor for PH, interstitial lung disease^10^ as well as a marker for low functional capacity by the Duke Activity Status Index,^11^ this study's findings revealed that abnormal RVSP was not a reliable marker for 30-day PPC in PH populations. Interestingly, RVSP provided incremental enhancement of ARISCAT in hybrid classification plots, suggesting improved prediction of 30-day PPC, PNA, and ASP but not RF in patients with RVSP < 40 mmHg. RVSP likely provides a snapshot of overall right heart health, whereas surgical stress requires more dynamic, individualized measures to predict vulnerability to respiratory insufficiency and failure. Static RVSP values may overlook the pulsatile nature of right ventricular work and its complex interactions with postoperative hemodynamic shifts, such as fluid overload, which underlies a large number of postoperative respiratory insufficiency/failure events. Alternative measures, such as pulmonary vascular impedance, warrant investigation as more sensitive markers for PPC vulnerability in PH populations. Recent publications have shown the predictive strength of measured pulmonary capacitance for prognosis quantification in PH and CHF, suggesting that it may be a compelling investigational target for PPC risk prediction.^12^^,^^13^ Interestingly, patients with RHC within 24-months of procedure were younger, had higher Elixhauser scores, but lower preoperative ARISCAT scores (p < 0.001). Although the significance is unclear, it suggests that these patients were better optimized prior to surgical procedures, given that there were no significant differences in PPC outcomes.

While higher ARISCAT score was associated with 30-day PPC, its clinical predictive strength remained limited for the robust AHRQ-PPC composite. The original ARISCAT focused on in-hospital PPC events.^14^ In contrast, the AHRQ-PPC composite includes important diagnoses such as pulmonary embolism, pneumonia, enhanced coding for respiratory failure/insufficiency, and extends PPC capture from procedure date through thirty days. The AHRQ-PPC composite offers broader complication capture, which is particularly important given the contemporary trend of shorter LOS. Despite this, sub composite analysis incorporating RVSP showed only modest improvement, failing to reach clinically significant levels (AUC > 0.8) despite all iterations showing modest improvement. Classification plots including hybridized ARISCAT+RVSP did demonstrate variable predictive power, when divided by RVSP thresholds. RVSP < 40 patients demonstrated improved PPC risk prediction using the hybridized ARISCAT+RVSP, while RVSP ≥ 40 prediction was diminished. The findings suggest that while current risk assessment tools provide valuable insights, there remains a significant opportunity to develop more robust, PH-specific predictive models that account for the complex pathophysiology of this patient population.

### Limitations

The study's findings have several factors that limit generalizability, including (1) Selection bias due to exclusion of patients with missing data, (2) Echocardiographic RVSP measurement variability, including its dependence on adequate tricuspid regurgitation and technical proficiency, and (3) Unmeasured confounding factors. The study population comprised of patients with ICD-10 coding of PH, highlighting the potential for inaccurate diagnosis. Out of 277 patients in this study, 93 underwent RHC within 24 months of surgery, and 87 of those 93 patients had mPAP > 20 mmHg. This implied a partial but reasonable positive predictive value for ICD-10 coding; however, it still limits the generalizability of the findings.

## Conclusion

RVSP demonstrated limited efficacy as a standalone predictor of 30-day PPC in patients with PH. Although integrating RVSP with ARISCAT scoring yielded marginal improvements in predictive accuracy, neither metric, independently or in combination, achieved adequate clinical significance for reliable risk stratification. These findings highlight a critical gap in the current preoperative risk assessment for PH-specific predictive tools. Future research should focus on alternative measures that better capture vulnerability to the hemodynamic complexities underscoring PPC in this high-risk population.

## Institutional Research Board Approval

Yale University Institutional Review Board #2000032516.

## Funding

Zyad J. Carr, M.D. receives research grant funding from Shape Medical Systems, Inc., Minnesota, USA.

## Authors’ contributions

Yoshio Tatsuoka: Study conception and design; data collection; results interpretation, manuscript writing and editing.

Hugo He: Statistical planning and analysis; results interpretation; manuscript writing and editing.

Hung-Mo Lin: Statistical planning and analysis; results interpretation; manuscript writing and editing.

Andrew P. Notarianni: Results interpretation; manuscript writing and editing.

Zyad J. Carr: Study conception and design; data collection; results interpretation, manuscript writing and editing.

## Declaration of competing interest

The authors declare no conflicts of interest.

## Appendix Supplementary materials

Image, application 1

## Footnotes

Supplementary material associated with this article can be found in the online version at doi:10.1016/j.bjane.2025.844597.
