# Supplementary material for: The role of right ventricular systolic pressure and ARISCAT score in perioperative pulmonary risk assessment
Source: Braz J Anesthesiol. 2025 Feb 17;75(2):844597. doi: 10.1016/j.bjane.2025.844597 (PMC11914786; doi:10.1016/j.bjane.2025.844597)
Supplement: Supplementary file 1 [file mmc1.docx]

**BJAN-D-24-00380_Supplementary Material**

**Supplementary Table 1** Definition of postoperative pulmonary complications and its sub composites of respiratory failure, infectious pneumonia, aspiration, and thromboembolic events, using International Classification of Disease version 10 codes provided by the Agency for Healthcare Research and Quality.

**Supplementary Table 1** Agency for healthcare research and quality 30-day postoperative pulmonary complication composite variables.

| **Variable** | **ICD-10** | **Subcomposite** |
| --- | --- | --- |
| Transfusion-related acute lung injury | J95.84 | AHRQ-RF |
| Respiratory complications not elsewhere classified | J95.85 | AHRQ-RF |
| Ventilator associated pneumonia | J95.86 | AHRQ-PNA |
| Post-procedural aspiration pneumonia | J95.87 | AHRQ-ASP |
| Other complication of ventilator | J95.88 | AHRQ-RF |
| Other intraoperative complications of respiratory system, not elsewhere classified | J95.89 | AHRQ-RF |
| Other postprocedural complications and disorders of respiratory system, not elsewhere classified | J95.90 | AHRQ-RF |
| Acute respiratory failure, unspecified whether with hypoxia or hypercapnia | J95.91 | AHRQ-RF |
| Respiratory failure, unspecified, unspecified whether with hypoxia or hypercapnia | J95.92 | AHRQ-RF |
| Respiratory failure, unspecified with hypoxia | J95.93 | AHRQ-RF |
| Respiratory failure, unspecified with hypercapnia | J95.94 | AHRQ-RF |
| Acute and chronic respiratory failure, unspecified whether with hypoxia or hypercapnia | J95.95 | AHRQ-RF |
| Acute and chronic respiratory failure with hypoxia | J95.96 | AHRQ-RF |
| Acute and chronic respiratory failure with hypercapnia | J95.97 | AHRQ-RF |
| Respiratory disorders in diseases classified elsewhere | J95.98 | AHRQ-RF |
| Pulmonary insufficiency following trauma and surgery | J95.99 | AHRQ-RF |
| Acute postprocedural respiratory failure | J95.100 | AHRQ-RF |
| Acute respiratory failure with hypoxia | J95.101 | AHRQ-RF |
| Acute respiratory failure with hypercapnia | J95.102 | AHRQ-RF |
| Acute pulmonary insufficiency following thoracic surgery | J95.103 | AHRQ-RF |
| Acute pulmonary insufficiency following nonthoracic surgery | J95.104 | AHRQ-RF |
| Chronic pulmonary insufficiency following surgery | J95.105 | AHRQ-RF |
| Acute and chronic postprocedural respiratory failure | J95.106 | AHRQ-RF |
| Acute respiratory distress syndrome | J95.107 | AHRQ-RF |
| Chronic respiratory failure, unspecified whether with hypoxia or hypercapnia | J95.108 | AHRQ-RF |
| Chronic respiratory failure with hypoxia | J95.109 | AHRQ-RF |
| Chronic respiratory failure with hypercapnia | J95.110 | AHRQ-RF |
| Respiratory arrest | J95.111 | AHRQ-RF |
| Pneumonia due to streptococcus pneumonia | J95.112 | AHRQ-PNA |
| Other bacterial pneumonia | J95.113 | AHRQ-PNA |
| Pneumonia due to Pseudomonas | J95.114 | AHRQ-PNA |
| Pneumonia due to streptococcus | J95.115 | AHRQ-PNA |
| Pneumonia due to staphylococcus | J95.116 | AHRQ-PNA |
| Pneumonia due to Methicillin susceptible Staphylococcus | J95.117 | AHRQ-PNA |
| Pneumonia due to Methicillin susceptible Staphylococcus aureus | J95.118 | AHRQ-PNA |
| Pneumonia due to Escherichia coli | J95.119 | AHRQ-PNA |
| Pneumonia due to other Gram-negative bacteria | J95.120 | AHRQ-PNA |
| Pneumonia due to other specified bacteria | J95.121 | AHRQ-PNA |
| Unspecified bacterial pneumonia | J95.122 | AHRQ-PNA |
| Pneumonia due to other specified infectious organisms | J95.123 | AHRQ-PNA |
| Invasive pulmonary aspergillus | J95.124 | AHRQ-PNA |
| Bronchopneumonia, unspecified organism | J95.125 | AHRQ-PNA |
| Pneumonia, unspecified organism | J95.126 | AHRQ-PNA |
| Pneumonia due to Klebsiella pneumonia | J95.127 | AHRQ-PNA |
| Pneumonia due to other streptococci | J95.128 | AHRQ-PNA |
| Pneumonitis due to solids and liquids | J95.129 | AHRQ-ASP |
| Hypostatic pneumonia, unspecified organism | J95.130 | AHRQ-PNA |
| Other ill-defined and unknown causes of morbidity and mortality | J95.131 |  |
| Respiratory conditions due to chemical fumes and vapors | J95.132 |  |
| Pneumonitis due to solids and liquids | J95.133 | AHRQ-ASP |
| Pneumonitis due to inhalation of food and vomit | J95.134 | AHRQ-ASP |
| Postprocedural pneumothorax | J95.135 |  |
| Septic pulmonary embolism without acute cor pulmonale | J95.136 | AHRQ-PE |
| Other pulmonary embolism without acute cor pulmonale | J95.137 | AHRQ-PE |
| Air embolism following infusion, transfusion and therapeutic injection, initial encounter | J95.138 | AHRQ-PE |
| Complication of other artery following a procedure, not elsewhere classified, initial encounter | J95.139 |  |
| Complication of vein following a procedure, not elsewhere classified, initial encounter | J95.140 |  |
| Embolism due to cardiac prosthetic devices, implants and grafts, initial encounter | J95.141 | AHRQ-PE |
| Embolism due to cardiac prosthetic devices, implants and grafts, initial encounter | J95.142 | AHRQ-PE |
| Embolism due to vascular prosthetic devices, implants and grafts, initial encounter | J95.143 | AHRQ-PE |
| Embolism due to vascular prosthetic devices, implants and grafts, initial encounter | J95.144 | AHRQ-PE |

AHRQ, Agency for Healthcare Research and Quality; PE, Pulmonary Embolism; RF, Respiratory Failure; ASP, Aspiration; PNA, Pneumonia.

**Supplementary Table 2** Unadjusted receiver operating characteristic area under curve estimates for the 30-day primary and secondary endpoints.

| **Outcome** | **Variable** | **AUC** | | | **RHC w/in 24 months** |
| --- | --- | --- | --- | --- | --- |
|  |  | **All** | **RVSP < 40** | **RVSP ≥ 40** |  |
| Postoperative pulmonary complications | RVSP | 0.555 | 0.658 | 0.519 | 0.501 |
|  | ARISCAT | 0.575 | 0.542 | 0.609 | 0.575 |
|  | RVSP + ARISCAT | 0.591 | 0.658 | 0.614 | 0.571 |
| Respiratory failure/insufficiency | RVSP | 0.573 | 0.542 | 0.557 | 0.567 |
|  | ARISCAT | 0.602 | 0.585 | 0.606 | 0.609 |
|  | RVSP + ARISCAT | 0.629 | 0.591 | 0.622 | 0.647 |
| Pneumonia | RVSP | 0.507 | 0.536 | 0.552 | 0.648 |
|  | ARISCAT | 0.555 | 0.626 | 0.605 | 0.538 |
|  | RVSP + ARISCAT | 0.552 | 0.644 | 0.611 | 0.662 |
| Aspiration events | RVSP | 0.542 | 0.577 | 0.564 | 0.578 |
|  | ARISCAT | 0.517 | 0.444 | 0.511 | 0.663 |
|  | RVSP + ARISCAT | 0.539 | 0.567 | 0.562 | 0.660 |
| Thromboembolic events | RVSP | 0.617 | 0.773 | 0.633 | 0.486 |
|  | ARISCAT | 0.524 | 0.873 | 0.633 | 0.604 |
|  | RVSP + ARISCAT | 0.604 | 0.904 | 0.658 | 0.633 |

RHC, Right Heart Catheterization; RVSP, Right Ventricular Systolic Pressure; ARISCAT, Assess Respiratory Risk in Surgical Patients in Catalonia risk index; AUC, Area Under the Curve.

**Supplementary Table 3** Baseline characteristics of the studied cohort categorized by presence or absence of right heart catheterization data within 24-months of procedure.

| **Variable** | **No RHC**  **(n = 190)** | **RHC**  **(n = 87)** | **p-value^a^** | **Total**  **(n = 277)** |
| --- | --- | --- | --- | --- |
| Age, in years, median (SD) | 68 (14.9) | 62.9 (12.8) | 0.004^a^ | 66.4 (14.5) |
| Gender, Female, number (%) | 114 (60) | 52 (59.8) | 1 | 166 (59.9) |
| Body Mass Index, kg.m^-2^, mean (SD) | 29.3 (8.81) | 29.5 (9.25) | 0.843 | 29.4 (8.9) |
| Elixhauser Comorbidities, mean (SD) | 6.19 (2.01) | 6.80 (1.93) | 0.017 | 6.39 (2) |
| Procedural Severity, number (%) |  |  |  |  |
| Intermediate | 112 (58.9) | 61 (70.1) |  | 173 (62.5) |
| Major | 39 (20.5) | 16 (18.4) |  | 55 (19.9) |
| Xmajor/Complex | 39 (20.5) | 10 (11.5) | 0.13 | 49 (17.7) |
| RVSP, in mmHg, mean (SD) | 51.3 (16.8) | 53.7 (18.8) | 0.319 | 52.1 (17.4) |
| ARISCAT score, mean (SD) | 31.3 (16.3) | 21.1 (16.3) | <0.001^a^ | 28.1 (17) |
| Surgical Time, in minutes, median (IQR) | 95 (52‒180) | 83 (44‒130) | 0.182 | 92.5 (51‒170) |
| PPC, number (%) | 55 (28.9) | 28 (32.2) | 0.686 | 83 (29.9) |
| Infectious pneumonia | 25 (13.2) | 9 (10.3) | 0.644 | 34 (12.3) |
| Respiratory failure | 35 (18.4) | 19 (21.8) | 0.612 | 54 (19.5) |
| Aspiration | 10 (5.3) | 5 (5.7) | 1 | 15 (5.4) |
| Pulmonary embolism | 7 (3.7) | 3 (3.4) | 1 | 10 (3.6) |
| Length of stay, in days, median (IQR) | 6.16 (3.3‒13.1) | 8.3 (4.2‒17.7) | 0.04 | 6.9 (3.7‒13.8) |
| Thirty-day Mortality, number (%) | 7 (3.7) | 4 (4.6) | 0.72 | 11 (3.9) |

^a^ Student’s *t*-test, Chi-square or Wilcoxon as appropriate.

RHC, Right Heart Catheterization; SD, Standard Deviation; RVSP, Right Ventricular Systolic Pressure by echocardiography; ARISCAT, Assess Respiratory Risk in Surgical Patients in Catalonia risk index; PPC, Postoperative Pulmonary Complications; IQR, Interquartile Range.
